# A cost-effectiveness analysis of pembrolizumab with or without chemotherapy for the treatment of patients with metastatic, non-squamous non-small cell lung cancer and high PD-L1 expression in Switzerland

**DOI:** 10.1007/s10198-021-01282-4

**Published:** 2021-03-21

**Authors:** Michaela Carla Barbier, Esther Pardo, Cédric Michael Panje, Oliver Gautschi, Judith Eva Lupatsch, for the Swiss Group for Clinical Cancer Research (SAKK)

**Affiliations:** 1 Institute of Pharmaceutical Medicine (ECPM), University of Basel, Klingelbergstrasse 61, 4056 Basel, Switzerland; 2 Medical Oncology, Department of Internal Medicine, Cantonal Hospital Lucerne, Lucerne, Switzerland; 3 Department of Radiation Oncology, Cantonal Hospital St. Gallen, St. Gallen, Switzerland; 4 University of Bern, Bern, Switzerland

**Keywords:** Non-small cell lung cancer, Pembrolizumab, Cost-effectiveness, Markov model, I19

## Abstract

**Introduction:**

Pembrolizumab monotherapy or in combination with chemotherapy are two new treatment options for patients with metastatic non-squamous non-small cell lung cancer (NSCLC) and high (≥ 50%) programmed death ligand 1 (PD-L1) expression. We conducted a cost-effectiveness analysis for Switzerland comparing these two options but also pembrolizumab to chemotherapy.

**Methods:**

We constructed a 3-state Markov model with a time horizon of 10 years. Parametric functions were fitted to Kaplan–Meier overall survival (OS) and progression-free survival (PFS) using 2-year follow-up data from the KN-024 and KN-189 registration trials. We included estimated costs for further treatment lines and costs for best supportive care. Costs were assessed from the Swiss healthcare payer perspective. We used published utility values.

**Results:**

Combination therapy resulted in an expected gain of 0.17 quality-adjusted life years (QALYs) per patient and incremental costs of Swiss Francs (CHF) 81,085 as compared to pembrolizumab. These estimates led to an incremental cost-effectiveness ratio (ICER) of CHF 475,299/QALY. Pembrolizumab in comparison to chemotherapy was estimated to generate mean incremental QALYs of 0.83 and incremental costs of CHF 56,585, resulting in an ICER of CHF 68,580/QALY. Results were most sensitive to changes in costs of 1L pembrolizumab and combination therapy, together with changes in PFS. In the probabilistic sensitivity analysis, we estimated combination therapy was cost-effective in 4.9% of the simulations and pembrolizumab monotherapy in 82.9%, assuming a willingness-to-pay threshold of CHF 100,000 per QALY gained.

**Conclusions:**

Pembrolizumab is likely to be cost-effective from the Swiss healthcare payer perspective, whereas pembrolizumab plus chemotherapy is not.

**Supplementary Information:**

The online version contains supplementary material available at 10.1007/s10198-021-01282-4.

## Introduction

Lung cancer is the second most common cancer and the most frequent cause of death among all cancers worldwide [1]. Non-small cell lung cancer (NSCLC) comprises 80–90% of lung cancer cases [2], with a strong predominance of non-squamous histology [3].

Treatment options for patients with metastatic NSCLC have evolved significantly over the last years with the implementation of molecular testing, targeted therapy, and immune-checkpoint inhibitors blocking the programmed cell death (ligand 1) (PD-L1/PD-1) pathway [3]. Among the checkpoint inhibitors approved by the United States (US) Food and Drug Administration (FDA) for the treatment of patients with metastatic NSCLC (nivolumab, pembrolizumab, and atezolizumab), pembrolizumab is the one that is most advanced, and therefore widely used, in the first-line (1L). For the treatment of NSCLC with high (≥ 50%) PD-L1 expression, pembrolizumab is approved as a single agent, based on the Keynote (KN)-024 trial [4]. In a recent retrospective analysis, Aguilar [5] deduced that among patients with PD-L1 ≥ 50%, 1L pembrolizumab was particularly beneficial for treating patients with PD-L1 ≥ 90%. Based on the KN-189 trial [6], pembrolizumab in combination with chemotherapy (combination therapy) is superior to chemotherapy monotherapy in patients with non-squamous NSCLC unselected by PD-L1 staining, and no sensitizing epidermal growth factor receptor (EGFR) or anaplastic lymphoma kinase (ALK) mutations. Due to the lack of data from a randomized trial directly comparing the combination therapy to pembrolizumab monotherapy, uncertainty remains about the added benefit of chemotherapy from clinical and economic perspectives. Insinga et al. [7] projected that combination therapy versus pembrolizumab monotherapy may potentially be cost-effective in the US for patients with PD-L1 ≥ 50% based on an indirect treatment comparison. Our aim was to carry out a cost-effectiveness analysis (CEA) of 1L therapies (chemotherapy, chemotherapy plus pembrolizumab, and pembrolizumab monotherapy) currently approved by the Swiss Agency for Therapeutic Products (Swissmedic) for the treatment of patients with metastatic non-squamous NSCLC and PD-L1 expression ≥ 50%, based on recent data from the KN-024 and KN-189 registration trials [4, 6].

## Materials and methods

### Model structure

We developed a 3-state Markov cohort simulation model with mutually exclusive health states of progression-free survival (PFS), progressive disease (PD) and death. The model was programmed in TreeAge software (Version 2019 R1.1) (supplementary Fig. S1). In TreeAge, second-line (2L) treatment was implemented with the help of tunnels.

We chose a model cycle length of 1 month applying half-cycle correction and a time horizon of 10 years based on the poor prognosis of stage IV NSCLC patients and the lack of more extended follow-up data for immunotherapy. An annual discount rate of 3% for costs and quality-adjusted life-years (QALYs) was applied.

Incremental cost-effectiveness ratio (ICER) of the pembrolizumab combination strategy versus pembrolizumab monotherapy and of pembrolizumab monotherapy versus chemotherapy were assessed in Switzerland, expressed as costs [in Swiss Franc (CHF)] per QALY gained.

### Population

We modelled a patient population with the characteristics of the KN-024 and KN-189 registration trials comprising of adult patients with previously untreated stage IV, mainly non-squamous NSCLC (100% non-squamous in KN-189, 81.6% in KN-024), without EGFR or ALK alterations. In KN-024, only patients with PD-L1 expression ≥ 50% had been recruited [8]. Since in KN-189, PD-L1 expression was not limited, we only used the subpopulation of patients with PD-L1 expression ≥ 50% [8, 9].

### Interventions

In line with the interventions of the aforementioned trials, we compared three treatment strategies: combination strategy (1L pembrolizumab plus chemotherapy, followed by 2L treatment with chemotherapy and best supportive care (BSC) or BSC directly), pembrolizumab strategy (1L pembrolizumab, followed by 2L chemotherapy and BSC or BSC directly), and chemotherapy strategy (1L chemotherapy, followed by 2L pembrolizumab and BSC or BSC directly) (supplementary Table S1, supplementary Methods section S1 Treatments).

Following discontinuation of 1L treatment, the percentage of patients as reported in each trial were assumed 2L treatment or BSC directly. Patients receiving 2L treatment were simulated to receive docetaxel (after 1L combination) for 4 cycles (= 2.76 months), platinum-based chemotherapy (carboplatin + paclitaxel, carboplatin + pemetrexed) for 4 cycles (after 1L pembrolizumab), followed by pemetrexed maintenance therapy for up to 35 cycles or until disease progression (only for patients on 2L pemetrexed), or 2L pembrolizumab until progression (after 1L chemotherapy). Patients were simulated to progress after either 6 cycles (4.2 months for 2L chemotherapy) or after 5.2 months (for 2L pembrolizumab) and to receive BSC, based on PFS results from 2L treatment studies [10] (supplementary Table S1). Where information for 1L and 2L treatments from the trials was available, we used the trial information. In case of missing or unclear information, we complemented or simplified information from the trials with our own clinical assumptions (adapted to the standard of care in Switzerland).

### Clinical parameters

#### Survival curve modelling

The Kaplan–Meier (KM) curves from 2-year follow-up data of the KN-024 [4] and the KN-189 [6] trials were converted to numeric values through digitalisation using the software application “DigitizeIt” [11]. Only for the PFS curve of the KN-024 trial, the original 1-year curve was digitalised since no updated PFS results were available [8]. We selected the survival curves with the best fit for PFS and OS respectively based on the Akaike and Bayesian information criterion (AIC, BIC), but also based on visual inspection of the closeness of the parametric curves to the 2-year KM plots and realistic long-term tails of the 10-year extrapolated survival curves. The distributions selected with the best fit were exponential distributions for all OS curves (supplementary Fig. S2) and lognormal distributions for all PFS curves (supplementary Fig. S3). Since Cox proportional hazard (PH) assumptions of the estimated survival curves were not met, we did not apply an indirect treatment comparison across the chemotherapy arms of both studies but used the trial arms individually. The chemotherapy arm of KN-189 was not further considered since its results for patients with high PD-L1 (≥ 50%) were very similar to the outcomes of the chemotherapy arm of KN-024 [4, 6] (supplementary Fig. S2 and S3). Estimated survival curves were converted into transition probabilities as one minus the ratio of the survivor function at the end and the beginning of a cycle. More information about survival curve modelling is available in supplementary Methods section S2 Survival curve modelling.

#### Adverse events

The model considered the occurrence of all reported grade 3 to 4 adverse events (AEs) from KN-024 [9] and KN-189 [3]. We separated pneumonitis, anaemia, colitis, exanthema (KN-024) and rash (KN-189), and summarized the remaining AEs in a category “other grade 3–4 toxicities”.

#### Utilities

We used published utilities (QALYs) for PFS under 1L treatment as provided by Huang [12], and utility values as reported by the National Institute for Health and Care Excellence (NICE) health in its technology assessment (HTA) report for progression-free disease, stratified by 2L treatment [13] (supplementary Table S2). More information can be found in supplementary Methods section S3 Utilities.

### Costs

We assessed costs from a Swiss healthcare payer perspective, where all direct medical costs were considered. Costs included regimen costs (drug costs), application costs (e.g. intravenous administration), diagnostic costs (e.g. computer tomography (CT) scans), costs for AEs of 1L treatment, and costs for other consumables and clinical visits. We detailed treatment costs during PFS and PD in supplementary Methods section S4 and supplementary Table S3.

### Uncertainty

To investigate parameter and structural uncertainty, we performed one-way and probabilistic sensitivity analyses, as well as several scenario analyses. For the sensitivity analyses, we varied 33 individual parameters (exponential and lognormal survival curve parameters, costs and utilities, discount rate, the probability distribution of 2L treatment after 1L chemotherapy and combination therapy) and presented the 15 most influential parameters in tornado diagrams. More details about the sensitivity analyses are given in supplementary section S5.

In a first scenario analysis, we assumed lower pemetrexed costs (as outlined in supplemental Table S3) based on the price of the cheapest available generic product in the United Kingdom (UK) but not available in Switzerland. We used the price specified by the British National Formulary and converted it into Swiss Francs [14]. In a further scenario analysis, we used the OS curve of the 1L chemotherapy arm of KN-024 adjusted for cross-over as provided by Reck et al. [9], thereby assuming that 2L pembrolizumab treatment was not possible. All patients were assumed to receive BSC directly after progression. In scenario analyses three, four and five, we investigated discount rates of 0% and 5%, as well as a time horizon of 5 years.

Scenario analysis six investigated an unlimited pemetrexed maintenance until progression or death, lifting the restriction of maximum 35 cycles. Rather than assuming treatment termination with disease progression, scenario analysis seven used the time-on-treatment (ToT) curves of Insinga et al. [7] for the combination therapy (KN189) and the ToT curves from Huang et al. (KN24)[15] for both pembrolizumab and chemotherapy. To explore a non-constant risk of mortality, we investigated in scenario analysis eight a lognormal distribution instead of an exponential distribution for the OS curves for all three treatment strategies.

## Results

### Base-case cost-effectiveness model results

Over 10 years, combination therapy relative to pembrolizumab monotherapy was projected to result, after discounting, in an expected gain of 0.17 QALYs per patient and incremental costs of CHF 81,085, resulting in an ICER of CHF 475,299 per QALY gained. Discounted incremental life years (LYs) of 0.29 resulted in incremental costs per LYs gained of CHF 277,600. A comprehensive list of all base-case results is provided in Table 1. For completeness, we report the result of a third-way comparison of the combination therapy relative to chemotherapy, which produced an ICER of CHF 138,266 per QALY gained.

**Table 1:** Base-case results

	Chemo strategy	Pem strategy	Incr Pem versus Chemo strategy	Combi strategy	Incr Combi versus Chemo strategy	Incr Combi versus Pem strategy
**Total costs (CHF)**	**98,794**	**155,379**	**56,585**	**236,464**	**137,670**	**81,085**
Chemotherapy 1L costs (CHF)^a^	30,801					
Pembrolizumab 1L costs (CHF)^a^		102,342				
Pembrolizumab-chemotherapy-combination 1L costs (CHF)^a^				183,787		
2L chemotherapy costs for patients in the pembrolizumab strategies (CHF)		3,019		493		
2L pembrolizumab therapy costs patients in the chemotherapy only strategy (CHF)	16,296					
Best supportive care costs (CHF)	35,300	34,603		37,161		
End of life costs (CHF)	16,398	15,415		15,023		
LYs (undiscounted)	1.86	2.99	1.14	3.33	1.47	0.34
LYs	1.76	2.77	1.01	3.06	1.30	0.29
Time in progression-free state (months)	7.17	22.24	15.08	25.54	18.37	3.30
Time in progressive state (months)	15.10	13.69	− 1.41	14.42	− 0.68	0.73
Proportion of estimated deaths	99.5%	95.9%		94%		
**QALYs**	**1.04**	**1.87**	**0.83**	**2.04**	**1.00**	**0.17**
**ICER (cost per QALY)**			**68,580**		**138,266**	**475,299**
Cost per LY gained (discounted)			55,989		105,678	277,600

^a^1L treatment cost include cost of diagnostics, drug acquisition, drug administration and treatment of adverse events

All results are discounted unless otherwise indicated

*1L* first-line, *2L* second-line, *Chemo* chemotherapy*, CHF* Swiss Franc, *ICER* incremental cost-effectiveness ratio, *Incr* Incremental, *LY* life-year, *Pem* pembrolizumab, *QALY* quality-adjusted life-year

Pembrolizumab monotherapy compared to chemotherapy was projected to generate mean incremental QALYs of 0.83 per patient and mean incremental costs of CHF 56,585, resulting in an ICER of CHF 68,580 per QALY gained. Mean total costs of CHF 155,379 for the pembrolizumab monotherapy strategy were largely comprised of 1L pembrolizumab treatment costs (CHF 102,342). For the chemotherapy strategy, the mean total costs of CHF 98,794 were mostly driven by BSC costs (CHF 35,300), followed by 1L chemotherapy costs (CHF 30,801).

### Scenario analyses

Table 2 presents the results of scenario analyses. Using a lower pemetrexed price based on the hypothetical reduction in its future price from a generic version becoming available, resulted in an ICER of CHF 265,158 per QALY gained for the combination versus pembrolizumab strategy, and an ICER of CHF 81,719 per QALY gained for the pembrolizumab versus chemotherapy strategy.

**Table 2:** Scenario analyses results

	Chemo strategy	Pem strategy	Incr Pem versus Chemo strategy	Combi strategy	Incr Combi versus Pem strategy
*Base case*					
Costs (CHF)	98,794	155,379	56,585	236,464	81,085
QALYs	1.04	1.87	0.83	2.04	0.17
ICER (cost per QALY)			**68,580**		**475,299**
*Scenario 1 (low pemetrexed costs)*					
Costs (CHF)	86,590	154,016	67,425	199,251	45,236
QALY	1.04	1.87	0.83	2.04	0.17
ICER (cost per QALY)			**81,719**		**265,158**
*Scenario 2 (OS chemo strategy cross-over adjusted, implying no 2L Pem use)*					
Costs (CHF)	72,490	155,379	82,889	236,464	81,085
QALYs	0.78	1.87	1.09	2.04	0.17
ICER (cost per QALY)			**76,126**		**475,299**
*Scenario 3 (0% discount rate for costs and outcomes)*					
Costs (CHF)	103,190	162,568	59,378	246,292	83,724
QALYs	1.09	2.02	0.92	2.21	0.19
ICER (cost per QALY)			**64,309**		**431,381**
*Scenario 4 (5% discount rate for costs and outcomes)*					
Costs (CHF)	96,146	151,079	54,933	230,558	79,479
QALYs	1.01	1.78	0.77	1.94	0.16
ICER (cost per QALY)			**71,454**		**504,894**
*Scenario 5 (5 year time horizon)*					
Costs (CHF)	94,576	146,348	51,772	224,975	78,626
QALYs	1.00	1.60	0.60	1.70	0.10
ICER (cost per QALY)			**85,925**		**757,479**
*Scenario 6 (unlimited pemetrexed maintenance until progression or death)*					
Costs (CHF)	100,441	155,401	54,960	294,114	138,714
QALYs	1.04	1.87	0.83	2.04	0.17
ICER (cost per QALY)			**66,611**		**813,101**
*Scenario 7 (treatment duration with ToT)*					
Costs (CHF)	87,180	151,069	63,889	186,468	35,398
QALYs	1.04	1.87	0.83	2.04	0.17
ICER (cost per QALY)			**77,433**		**207,495**
*Scenario 8 (lognormal distribution for all OS curves)*					
Costs (CHF)	114,289	177,124	62,835	260,052	82,928
QALYs	1.27	2.34	1.07	2.46	0.12
ICER (cost per QALY)			**58,899**		**709,301**

*Chemo* chemotherapy*, CHF* Swiss Franc, *Comb*i combination, *ICER* incremental cost-effectiveness ratio, *Incr* Incremental, *OS* overall survival, *Pem* pembrolizumab, *QALY* quality-adjusted life-year, *ToT* time on treatment

When patients under 1L chemotherapy treatment were assumed not to be able to switch to 2L pembrolizumab, an ICER of CHF 76,126 per QALY gained was estimated for the comparison of pembrolizumab monotherapy versus chemotherapy. Using a shorter time horizon of 5 years resulted in higher estimated ICERs.

As expected, unlimited pemetrexed maintenance increased costs particularly for chemotherapy and combination strategies leading to an ICER of CHF 813,101 for the comparison of the combination and pembrolizumab groups. The final conclusions of the comparisons were however not affected. Use of ToT curves to approximate drug and administration costs only slightly increased the ICER for the comparison pemetrexed to chemotherapy. However, the ICER of the comparison of the combination strategy to pembrolizumab is approximately halved. Using a lognormal distribution to approximate and extrapolate the OS KM curves, resulted in an increased ICER of CHF 709,301 per QALY gained for the combination versus pembrolizumab strategy, and a similar ICER of CHF 58,899 per QALY gained for the pembrolizumab versus chemotherapy strategy. Again, the final conclusions of the comparisons were not affected.

### Sensitivity analysis

**Fig. 1:**
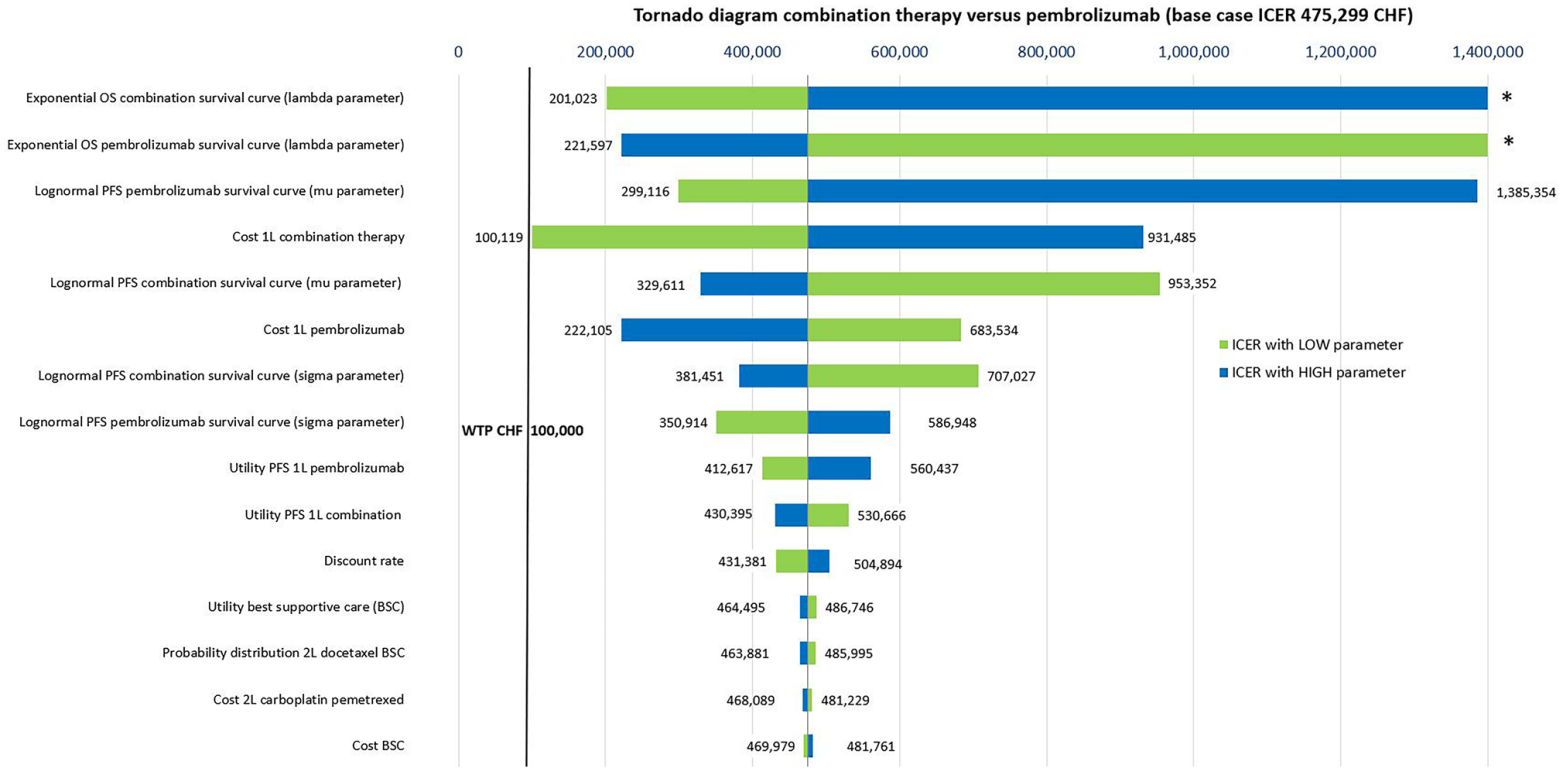
Tornado diagram of sensitivity analyses comparing 1L combination to pembrolizumab monotherapy strategy. *AEs* adverse events, *CHF* Swiss Franc, *ICER* incremental cost-effectiveness ratio, *PFS* progression-free survival, *WTP* willingness-to-pay threshold (CHF per QALY gained). (Asterisk) Combination strategy dominated

The tornado graph in Fig. 1 shows that variations in the parameter estimates of the modelled PFS curves as well as costs of the combination and the pembrolizumab therapies had the greatest impact on the ICER when comparing the combination relative to the pembrolizumab strategy. Reducing the cost of the combination therapy to the lower bound of its 95% confidence interval resulted in an ICER of CHF 100,119 per QALY gained. Variation in the OS (exponential) curve parameter for pembrolizumab reduced the ICER to CHF 221,597/QALY (high curve estimate) and for the combination strategy to CHF 201,023/QALY (low curve estimate), but also resulted in the combination strategy being dominated by pembrolizumab strategy (low estimate for combination and high estimate for pembrolizumab OS curve).

**Fig. 2:**
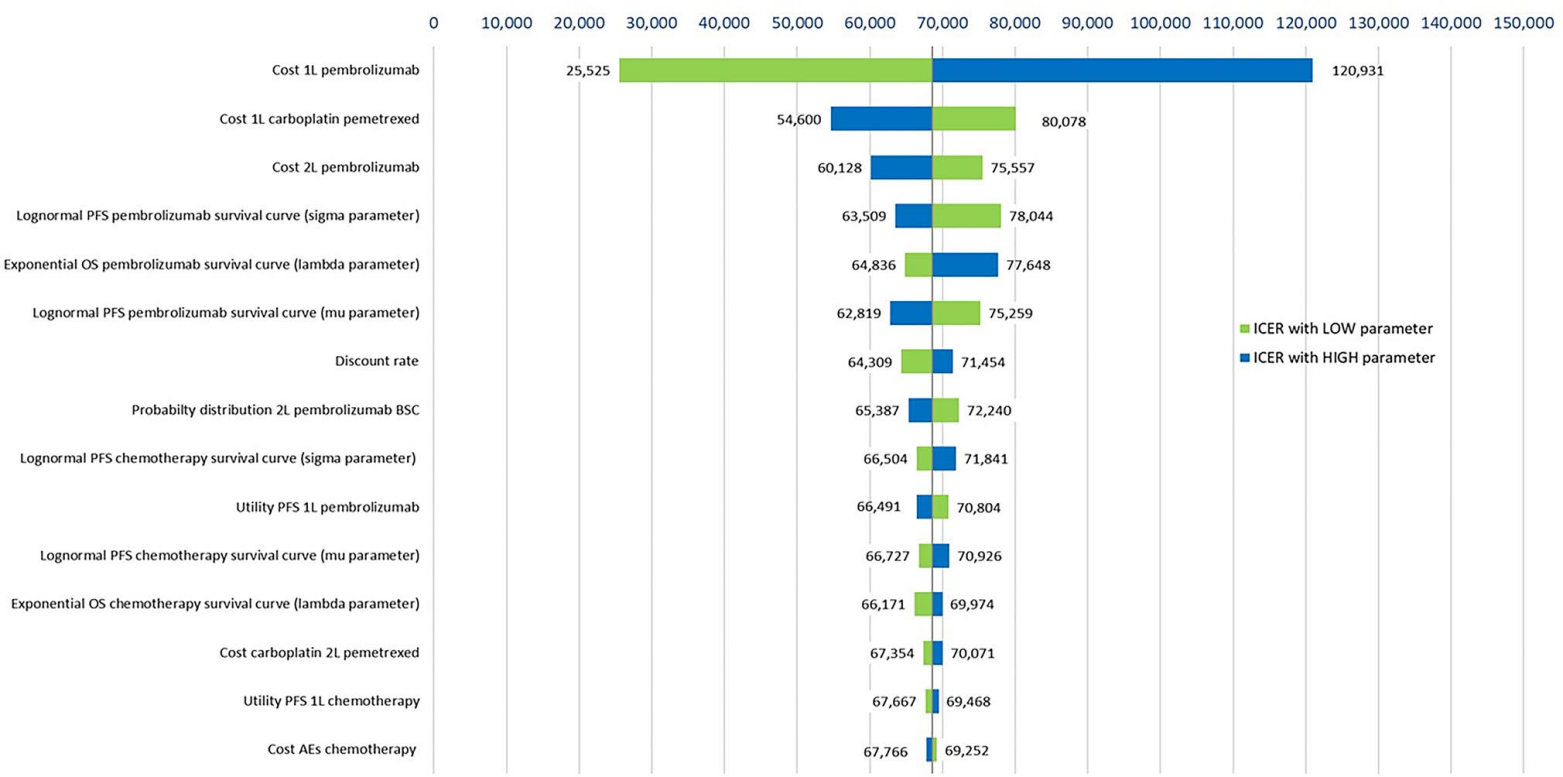
Tornado diagram of sensitivity analyses comparing 1L pembrolizumab to chemotherapy strategy. *AE* adverse event, *CHF* Swiss Franc, *ICER* incremental cost-effectiveness ratio, *OS* overall survival, *PFS* progression-free survival, *WTP* willingness-to-pay threshold (CHF per QALY gained)

For the comparison of the pembrolizumab strategy relative to the chemotherapy strategy (Fig. 2), variation in the cost of 1L pembrolizumab had the most substantial influence on the ICER.

In probabilistic sensitivity analyses, we estimated pembrolizumab to be cost-effective in 82.9% of the simulations and the combination therapy in 4.9% of the simulations, assuming a willingness-to-pay threshold (WTP) threshold of CHF 100,000 per QALY gained (supplementary Fig. S4 to S6).

## Discussion

Over a 10-year time horizon, we estimated mean improvements of 0.34 LYs (undiscounted) and 0.17 QALYs (discounted) per patient, together with an increase in mean costs of CHF 81,085, when comparing the combination strategy from KN-189 (induction with 1L pembrolizumab plus pemetrexed and carboplatin, followed by maintenance with pembrolizumab and pemetrexed) to the pembrolizumab monotherapy strategy from KN-024. The resulting ICER was CHF 475,299 per QALY gained. This suggests that combination therapy is not cost-effective in Switzerland if a threshold of CHF 100,000 per QALY gained is assumed. Results were robust to changes in the majority of the input parameters but were sensitive to changes in parameter estimates of the modelled PFS curves as well as in costs of the combination and the pembrolizumab therapies. High costs of 1L and 2L chemotherapy were partially driven by high Swiss costs of commercial pemetrexed. Currently, there is no pemetrexed generic approved in Switzerland. In the US, Insinga et al. [7] projected that combination therapy may potentially be cost-effective for patients with PD-L1 ≥ 50% based on an indirect treatment comparison and a threshold of 3-times the US per capita gross domestic product of US dollar (i.e. about USD 180,000 per QALY gained).

The cost-effectiveness of 1L pembrolizumab strategy relative to the chemotherapy strategy was CHF 68,580 per QALY gained, suggesting that pembrolizumab monotherapy is cost-effective in Switzerland. Over a 10-year time horizon, we estimated mean improvements of 1.14 LYs (undiscounted) and 0.83 QALYs (discounted) per patient, together with an increase in mean costs of CHF 56,585. Variation in the cost of 1L pembrolizumab had a substantial influence on the ICER. Assuming it was not possible for patients previously receiving 1L chemotherapy to switch to 2L pembrolizumab after disease progression, the estimated ICER increased to CHF 76,126 per QALY gained, but would still be cost-effective. The findings for the pembrolizumab versus chemotherapy comparison are in line with previous CEA results from the partitioned survival model by Huang et al. for the US [15] and its adaptation for France [16] and Switzerland [17]. Further studies which found 1L pembrolizumab is likely to be cost-effective based on different models were made for the US [18], Singapore [19] and Hong Kong [20]. In contrast, 1L pembrolizumab without a discount in its list price has been reported as potentially not cost-effective in China [21] and the UK [22].

One major strength of our analysis is the use of recently published 2-year survival data of the KN-024 [4] and KN-189 trials [6], and the fact that the pembrolizumab and the chemotherapy arms had been directly compared in the KN-024 trial. For the combination therapy, no NSCLC trials with high PD-L1 expression directly comparing combination therapy to pembrolizumab exist. However, as the Cox PH assumption of the estimated survival curves was not met (neither for KN-189 for OS nor for KN-024 for PFS), we did not apply an indirect treatment comparison across both studies. We used the trial arms individually. The chemotherapy arm of KN-189 was not considered further because its results for patients with PD-L1 ≥ 50% were very similar as in KN-024. In contrast to Huang et al. [15] we used parametric survival curves for the whole 10-year time horizon and did not apply a piecewise approach.

A further strength is that we also used published utilities for progression-free disease under 1L treatment [12], directly derived from the KN-024 and KN-189 trials. Utilities for progression-free disease under 2L treatments (pembrolizumab, docetaxel) were consistently taken from a related NICE HTA appraisal [13]. Another strength is that our analysis was conducted independently from the pharmaceutical industry.

There are some limitations to our analysis. Due to the independent modelling of survival curves, we could not directly vary the treatment effect in sensitivity analyses. Survival curves with the best fit were selected based on the AIC and BIC criteria in relation to the 2-year KM-curves and also based on visual inspection of the longer-term fit (10 years), assuming the majority of the patients being dead after 10 years. Extrapolations beyond the 2-year clinical effectiveness data may need adaptation when longer-term follow-up data become available. Recent evidence [7, 23] indicates that the mortality risk may be decreasing rather than being constant over time. For this reason, we investigated in an additional scenario analysis a lognormal distribution to estimate overall survival. The results did not change the overall conclusions of any comparison.

A further limitation is that a proportion of KN-024 patients had squamous NSCLC (18.4%). We were unable to exclude these patients from our analysis, as published PFS and OS results were not stratified by squamous versus non-squamous histology. Assumptions on 2L treatments and their duration were sourced from information provided in the KN-024 and KN-189 trials. In case of missing or unclear information these had to be complemented with own clinical assumptions. We only included cost of AEs due to 1L treatment and assumed that costs of AEs due to 2L treatment could be neglected.

In conclusion, Pembrolizumab monotherapy is likely to be cost-effective from the Swiss healthcare payer perspective, whereas pembrolizumab plus chemotherapy is not. INSIGNA (NCT03793179), and further trials comparing immunotherapy with combination immuno-chemotherapy, will allow validation of these findings, and in-depth analysis of NSCLC subsets characterized by PD-L1 in the near future.

## Supplementary Information

Below is the link to the electronic supplementary material.

Supplementary file1 (DOCX 522 KB)
