# Supplementary material for: A cost-effectiveness analysis of pembrolizumab with or without chemotherapy for the treatment of patients with metastatic, non-squamous non-small cell lung cancer and high PD-L1 expression in Switzerland
Source: Eur J Health Econ. 2021 Mar 21;22(5):669–77. doi: 10.1007/s10198-021-01282-4 (PMC8214587; doi:10.1007/s10198-021-01282-4)
Supplement: Supplementary file 1 — Supplementary file1 (DOCX 522 KB) [file 10198_2021_1282_MOESM1_ESM.docx]

**A cost-effectiveness analysis of pembrolizumab with or without chemotherapy for the treatment of patients with metastatic non-squamous non-small cell lung cancer and high PD-L1 expression in Switzerland**

The European Journal of Health Economics

Michaela Barbier*, Esther Pardo*, Cedric Panje, Oliver Gautschi, Judith E. Lupatsch, for the Swiss Group for Clinical Cancer Research (SAKK)

*shared first authorship

**Electronic Supplementary Materials**

**Supplementary Methods S1 Treatments**

Treatment regimens used for the model were consistent with their applications in the relevant trials, unless otherwise stated. In Keynote (KN)-024, pembrolizumab was administered as infusions of 200 mg intravenously every 3 weeks. Treatment was continued until disease progression or for a maximum of 35 cycles. The control group in KN-024 received first-line (1L) chemotherapy treatment. Possible chemotherapy regimens were carboplatin + pemetrexed, carboplatin + gemcitabine, carboplatin + paclitaxel, or cisplatin + gemcitabine. These were all administered for 4 cycles except for carboplatin + pemetrexed, in which pemetrexed was continued as maintenance therapy for up to 35 cycles or until disease progression. We restricted pemetrexed maintenance to maximum 35 cycles in the base case assuming all chemotherapy patients to have discontinued pemetrexed maintenance after 72 weeks [1].

In KN-189, combination therapy of pembrolizumab plus chemotherapy comprised of either cisplatin + pemetrexed or carboplatin + pemetrexed. In our model we deliberately omitted cisplatin + pemetrexed as a treatment option, as in Switzerland carboplatin + pemetrexed is favoured (shorter infusion times, lower neuro- and nephrotoxic side effect profile). Hence, for KN-189, 1L treatment with pembrolizumab plus chemotherapy was based on infusions of a standard dosage of pembrolizumab 200 mg intravenously every 3 weeks and of pemetrexed 500 mg/$m^{2}$, to which during the first 4 cycles carboplatin was added (dosage simplified to 400 mg/$m^{2}$ to avoid dose calculations based on kidney function) (all 3 given every 21 days). After conclusion of the chemotherapy cycles, the combination therapy of pembrolizumab 200 mg and pemetrexed 500 mg/$m^{2}$ every 21 days was continued for 31 subsequent cycles or until disease progression.

Regarding second-line (2L) treatment possibilities, at progression after or during 1L pembrolizumab, the option was 2L treatment with carboplatin 400 mg/$m^{2}$ combined with either pemetrexed 500 mg/$m^{2}$ (non-squamous histology) or paclitaxel 200 mg/$m^{2}$ (squamous histology) every 21 days for a mean of 4 cycles (2.76 months). For patients who received pemetrexed, chemotherapy was followed by maintenance therapy with pemetrexed 500 mg/$m^{2}$ every 21 days, again up to 35 cycles or until disease progression. Supplementary Table S1 shows the details. We further assumed a median progression-free survival (PFS) of 2L platinum based chemotherapy treatment of 6 cycles (4.2 months) as for docetaxel, based on Borghaei et al. 2015 [2], Matter-Walstra et al. 2016 [3], and Schiller et al. 2002 [4].

At progression after or during 1L chemotherapy, patients could switch to 2L immunotherapy with pembrolizumab. The percentage of patients in KN-024 who switched from chemotherapy to immunotherapy was estimated at 65.1% based on a study by Reck et al. [5] (supplementary Table S1). For patients who switched, we further assumed a median PFS under pembrolizumab of 5.2 months based on Herbst et al. [6], and modelled best supportive care (BSC) treatment afterwards. For 2L treatment after combination therapy, we assumed administration of docetaxel for a mean of 4 cycles for 32.6% of the patients (based on Gadgeel et al. updated results [7]) and BSC for the remaining 67.4%. We assumed again that patients under and after receiving docetaxel treatment remained progression-free during a median of 6 cycles and received BSC afterwards.

**Supplementary Methods S2 Survival curve modelling**

After digitalisation of the original Kaplan Meier (KM)-curves of KN-024 and KN-189, we applied the method of Guyot 2012 [8] to re-create the overall survival (OS) and PFS KM-curves and to estimate individual patient data with the help of R programs (R version 3.6.0; R Studio 1.2.1335, Version). In accordance with one possible modelling approach recommended by the National Institute of Health and Care Excellence (NICE) Decision Support Unit for fitting parametric models to survival curves, we tried to fit several survival curves (Exponential, Weibull, Logistic, Lognormal, Loglogistic, Gamma, Gompertz and Generalized Gamma) separately for each treatment arm to the re-created data [9].

**Supplementary Methods S3 Utilities**

For the combination strategy based on KN-189, we used the published utility of 0.768 during PFS for the whole KN-189 population and assumed the same utility for the subgroup of patients with programmed cell death ligand 1 (PD-L1)>=50%. This may represent a conservative approach, although Huang et al. [10] even concluded that only small utility differences were observed between PD-L1 ≥50% and the respective overall trial populations. In their opinion, utilities for specific health states did not vary substantially by PD-L1 status. Between cessation of 2L chemotherapy treatment and BSC treatment/progression in our model, we assumed the same utility as during 2L chemotherapy treatment. For progressive disease/BSC, a utility estimate of 0.47 was used, based on previous studies [3,11-13].

We assumed that any utility decrements associated with adverse events would have already been captured in the European Quality of Life 5 Dimensions (EQ-5D) scores from the above mentioned trials. Therefore no further utility decrements were applied to this model.

**Supplementary Methods S4 Costs**

Costs were calculated as cost per three-week treatment cycle (21 days) and normalised to the average length of one month (model cycle).

Drug costs were sourced from the official public prices of the Swiss specialty list [14]. If generic formulations were available, the price of the cheapest available formulation was used. Price was calculated per mass unit of a substance using the most used vial size. We applied a mean body surface area (mean 1.82$m^{2}$, 95% confidence interval (CI) [1.31, 2.36]) from a publication by Stene et al. whenever necessary [11]. For outpatient costs, we used the TARMED system (version 01.09.00_BR_KVG, accessed via BAG TARMED Browser) [15]. Costs of consumables were calculated by current standard of the cantonal hospital of Lucerne.

Treatment costs

During PFS a computer tomography (CT) scan was assumed every 9 weeks during a treatment phase (including maintenance therapies), while during a therapy-free phase the CT scan was only expected every 12 weeks.

After termination of treatment while in progression-free state (1L and 2L independent), monthly costs of Swiss francs (CHF) 337.69 arose during the first 24 months after treatment (including a routine visit with the oncologist every three months with routine laboratory testing and a CT scan of chest and abdomen), CHF 168.85 per month in the following 36 months (including a routine visit with the oncologist every sixth months with routine laboratory and a CT of chest and abdomen), and CHF 84.42 per month thereafter (including a yearly visit with the oncologist with routine laboratory testing and a CT scan of chest and abdomen).

Costs for adverse events

Costs for grade 3-4 adverse events (AEs) for 1L treatment were calculated based on the reported frequencies in KN-024 (Reck et al. [16]) and KN-189 (Gandhi et al. [5]). In line with the pembrolizumab NICE health technology assessment (HTA) [10], the impact of AEs was incorporated by estimating weighted average costs per patient, applied as a one-off cost (supplementary Table S4). These were then applied in the first cycle of the model for each treatment arm. We did not apply AE costs of 2L treatment.

Costs for grade 5 AEs (death) were included in the end of life (EOL) costs.

Costs for end of life care (one-time costs)

It is difficult to estimate ambulant care costs for terminal and EOL care costs. Not all patients die in hospital but the vast majority receives some hospital treatment at the end of life. We based our estimation on palliative care hospitalisation costs in the Swiss Cantonal Hospital of Lucerne in 2018. We assumed that the percentage of hospitalised patients for EOL care with lung cancer in Switzerland is 76% [17] resulting in one-off EOL costs of CHF 22,816*0.76 = CHF 17,340.16 per patient.

Costs for best supportive care

Costs for BSC in Switzerland were estimated at CHF 2,903 per month (supplementary Table S3) based on previously published data by Matter-Walstra in 2016 [3] with subsequent inflation adjustment to the year 2018 using the Swiss Consumer Price Index (inflation multiplier of 1.015 from 2016 to 2018) [18].

**Supplementary Methods S5 Uncertainty**

For the sensitivity analyses, we assigned gamma distributions to unit cost parameters and beta distributions to utilities and probabilities. The parameter estimates of the OS and PFS curves were assigned normal distributions. Available standard errors in combination with the applied distributions were used for the probabilistic sensitivity analysis (PSA) with 100,000 simulation runs. If there were no standard error estimates or 95% CIs available, we assumed standard errors to be 20% of the base case parameter values for costs and probabilities and 10% for utilities. For the univariate sensitivity analyses, individual parameters were varied by their 95% CIs.

**Supplementary Tables and Figures**

Table S1 Treatment lines and frequencies

| **Study** | **1L treatment** | **2L treatment** | **Percentage of 2L treatment** |
| --- | --- | --- | --- |
| KN-024 | Pembrolizumab monotherapy maximum 35 cycles | Carboplatin + Paclitaxel for a mean of 4 cycles (2.76 months). BSC only after median PFS of 4.2 months [2-4]. | 7.9% (18.8% squamous patients out of 42.2% [19]) |
|  |  | Carboplatin + Pemetrexed (mean 4 cycles) followed by pemetrexed maintenance (median PFS of 4.2 months). BSC directly after pemetrexed maintenance. | 34.3% (81.2% non-squamous patients out of 42.2%) |
|  |  | BSC | 57.8% |
| KN-024 | Carboplatin + Pemetrexed 68.2%, Carboplatin + Gemcitabine 13.2%, Carboplatin + Paclitaxel 11.3%, Cisplatin + Gemcitabine 7.3% [20]  4 cycles for all regimens except Carboplatin + Pemetrexed which is followed by Pemetrexed maintenance (up to cycle 35 cycles) | Pembrolizumab monotherapy for a median PFS of 5.2 months [6], then BSC. | 65.1 % [5] |
|  |  | BSC | 34.9% |
| KN-189 | Pembrolizumab + Carboplatin + Pemetrexed (4 cycles), followed by Pembrolizumab + Pemetrexed (31 cycles) | Docetaxel for a mean number of 4 cycles. BSC start after a median PFS of 4.2 months. | 32.6% [7] |
|  |  | BSC | 67.4% |

*BSC* best supportive care, *PFS* progression-free survival

Table S2 Utility summary table

| **Input parameter** | **Mean annual utility score** | **Variation (95% CI) for sensitivity analysis** | **Distribution for PSA** | **Reference** |
| --- | --- | --- | --- | --- |
| **Progression-free-disease state (First-line)** | | | | |
| **First-line** |  |  |  |  |
| Progression-free-survival under 1L pembrolizumab | 0.808 | (0.793, 0.823) | Beta | [10] |
| Progression-free-survival under 1L platinum-based chemotherapy | 0.757 | (0.738, 0.775) | Beta | [10] |
| Progression-free survival under 1L combined pembrolizumab chemotherapy | 0.768 | (0.759, 0.777) | Beta | [10] |
| **Progression-free-disease state (Second-line)** | | | | |
| 2L Pembrolizumab | 0.765 | (0.749,0.782) | Beta | [21] |
| 2L Docetaxel | 0.736 | (0.719-0.754) | Beta | [21] |
| **Progressive disease state** | | | | |
| Best supportive care (disease progression) | 0.47 | (0.378-0.561) | Beta | [3,11,12] |
| *1L* first-line, *2L* second-line | | | | |

Table S3 Treatment costs for progression-free and progressive disease state

| **Treatment** | **Drug cost per month** | **Other costs per month** |
| --- | --- | --- |
| **Progression-free disease** |  |  |
| **KN-024** |  |  |
| Carboplatin (400 mg/m^2 b^) and Paclitaxel (200 mg/m^2^), 4 cycles | First 2.76 months CHF 733.41  Afterwards (> 2.76 months): CHF 0 | First 2.76 months CHF 1,799.99  Afterwards (> 2.76 months)^d^:  0- 24 months: CHF 337.69  25 – 60 months: CHF 168.85  >60 months: CHF 84.42 |
| Carboplatin (400 mg/m^2 b^) and Pemetrexed (500 mg/m^2^), 4 cycles. Afterwards pemetrexed maintenance (500 mg/m^2^) until cycle 35 (2 years)^f^ | First 2.76 months: CHF 5,244.51  *Or generic price ^e^: CHF 3,008.72*  >2.76 months to 24 months (35 cycles): CHF 4,990.35  *Or generic price ^e^: CHF 2,050.24*  > 24 months: CHF 0  *Or >2.76 months: CHF 4,990.35^f^* | First 2.76 months: CHF 1,340.52  >2.76 months to 24 months (35 cycles): 711.13 CHF  > 24 months: ^d^  *Or >2.76 months: 711.13 CHF^f^* |
| Carboplatin (400 mg/m^2 b^) and Gemcitabine (1250 mg/m^2 c^), 4 cycles | For the first 2.76 months: CHF 961.42  Afterwards: CHF 0 | For the first 2.76 months: CHF 1,729.39  Afterwards (> 2.76 months): ^d^ |
| Cisplatin (5 mg/m^2 b^) and Gemcitabine (1250 mg/m^2 c^), 4 cycles | First 2.76 months: CHF 1,030.47  Afterwards: CHF 0 | First 2.76 months: 1900.96 CHF  Afterwards: ^d^ |
| Pembrolizumab (200mg flat), for maximum 25 cycles | First 24 months: CHF 7,566.36  Afterwards: CHF 0 | First 24 months: CHF 842  Afterwards: ^d^ |
| **KN-189 (Pembrolizumab combination arm)** | | |
| Pembrolizumab combination ^a^  (pembrolizumab 200mg flat dose + carboplatin 400mg/m2 + pemetrexed 500mg/m2), 4 cycles for all 3 drugs.  Afterwards: Up to 31 cycles Pembrolizumab + Pemetrexed^f^ | First 2.76 months: CHF 12,810.87  *Or generic price ^e^: CHF 10,575.08*  >2.76 months and up to 24 months (35 cycles): CHF 12,556.71  *Or generic price ^e^: CHF 9,616.60*  > 24 months: CHF 0  *> 24 months: CHF 4,990.35^f^* | First 2.76 months: CHF 1,499.61  >2.76 months and up to 24 months (35 cycles): CHF 918.63  > 24 months: ^d^  *> 24 months: 711.13 CHF^f^* |
| **Progressive disease** |  |  |
| Docetaxel (75 mg/m2), 4 cycles | First 2.76 months: CHF 912.10  Afterwards: CHF 0 | First 2.76 months: CHF 1,248.77  Afterwards: ^d^ |
| Pembrolizumab or Carboplatin Paclitaxel or Carboplatin Pemetrexed | As in progression-free state  Exception: Pembrolizumab second line not limited to 35 cycles, but given until progression. Hence at any time: CHF 7,566.36 | as in progression-free state  For Pembrolizumab at any stage: CHF 842 |
| BSC |  | CHF 2,903 |
| (1 Month = 30.44 days = 4.35 weeks, 1 year=52.2 weeks)  ^a^ Note: We assumed that only carboplatin but no cisplatin is used in Switzerland in this setting as described above.  ^b^ Simplified to a reasonable dose per m2 to avoid calculations of kidney function (eGFR).  ^c^ Gemcitabine is applied on day 1 and 8 at a dose of 1250mg per 3 week cycle.  ^d^ After termination of treatment, while in progression free state, the following monthly costs arise in CHF as previously mentioned: 0 - 24 months: CHF 337.69; 25 – 60 months: CHF 168.85; >60 months: CHF 84.42.  ^e^ Calculated with a generic pemetrexed (based on cheapest available generic product in UK according to bnf.nice.co.uk).  ^f^ In a scenario analysis we assumed pemetrexed maintenance therapy unlimited (until progression or death), pembrolizumab for a maximum of 35 cycles (if applied). | | |

*BSC* best supportive care, *CHF* Swiss Franc, *mg* milligram

Table S4 Grade 3-4 AEs probabilities for 1L AEs included in the economic model

| **Toxicity** | **Source** | **Costs per patient (in CHF)** | **Pembrolizumab**  **(in %)** | **Chemotherapy**  **(in %)** | **Pembrolizumab and chemotherapy (in %)** |
| --- | --- | --- | --- | --- | --- |
| Pneumonitis  CTC 3-4 | DRG E75C | 6,542.00 | 2.6 | 0.7 | 2.0 |
| Anaemia CTC 3-4 | TARMED | 1,597.31 | 1.3 | 19.3 | 16.3 |
| Colitis CTC 3-4 | Real world data | 24,464.00 | 1.9 | 0 | 0.0 |
| Exanthema/ Rash* CTC 3-4 | Real world data | 699.80 | 5.2 | 0 | 2.0 |
| Other grade 3-4 toxicities | DRG E71B | 3,853.5 | 18.9 | 31.3 | 40.2 |
| **Total %** | | | 29.9 | 51.3 | 60.5 |
| **Total weighted costs per patient (in CHF)** | | | **1,420** | **1,560** | **1,954** |
| *Note on KN-189, KN-024: AE data were collected differently in both studies. While in KN-024 diarrhoea and colitis were differentiated, in KN189 no colitis is mentioned although a quite higher rate of severe grading diarrhoea. Same has to be noted about dyspnoea versus pneumonitis. To be able to compare the costs of the AE’s, we treated the following under immunotherapy as follows: CTC 3-4 dyspnoea as pneumonitis and diarrhoea CTC 3-4 counting as a colitis, assuming the colitis may be underdiagnosed and the severe symptoms being in need of similar to same diagnostic workup. | | | | | |

*AE* adverse event, *CHF* Swiss Franc


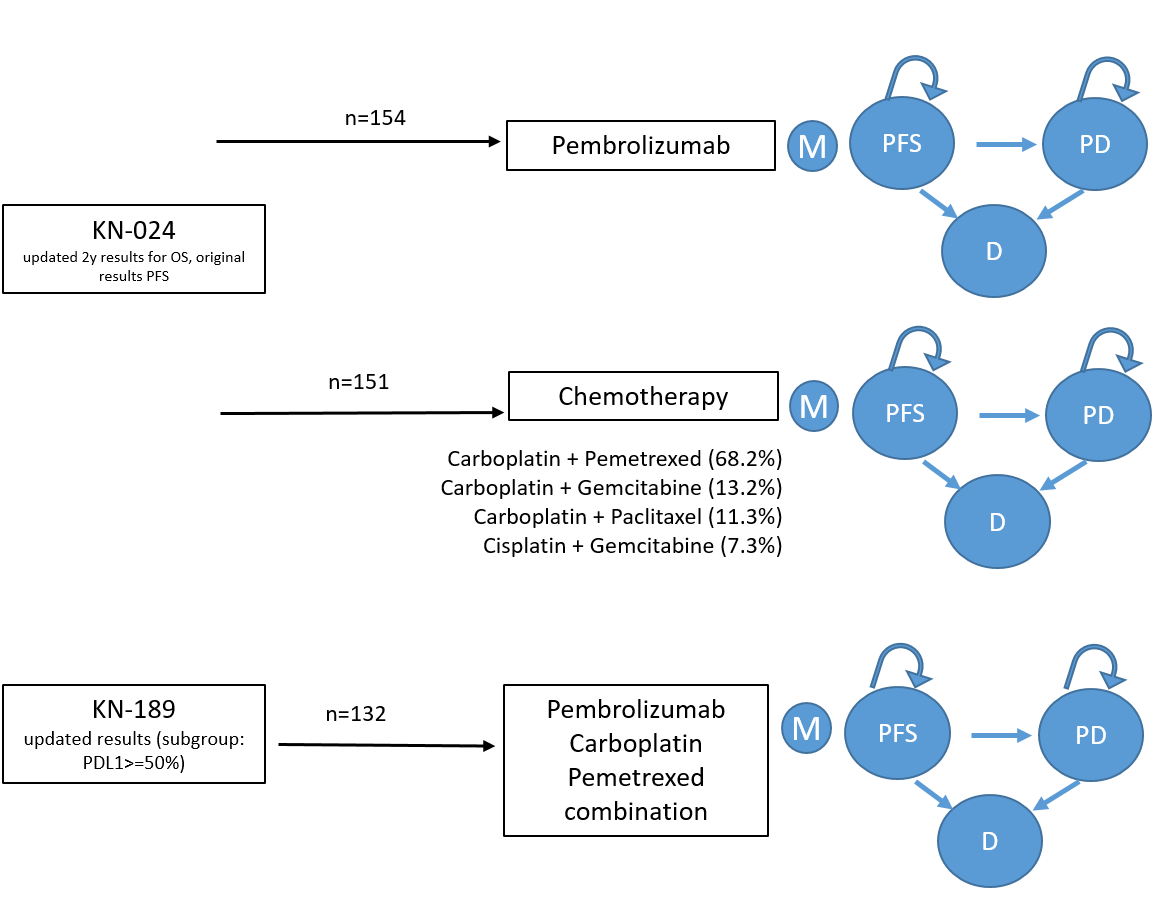


**Fig. S1** Markov model overview. *D* death, *M* Markov, *PD* progressive disease, *PFS* progression-free survival


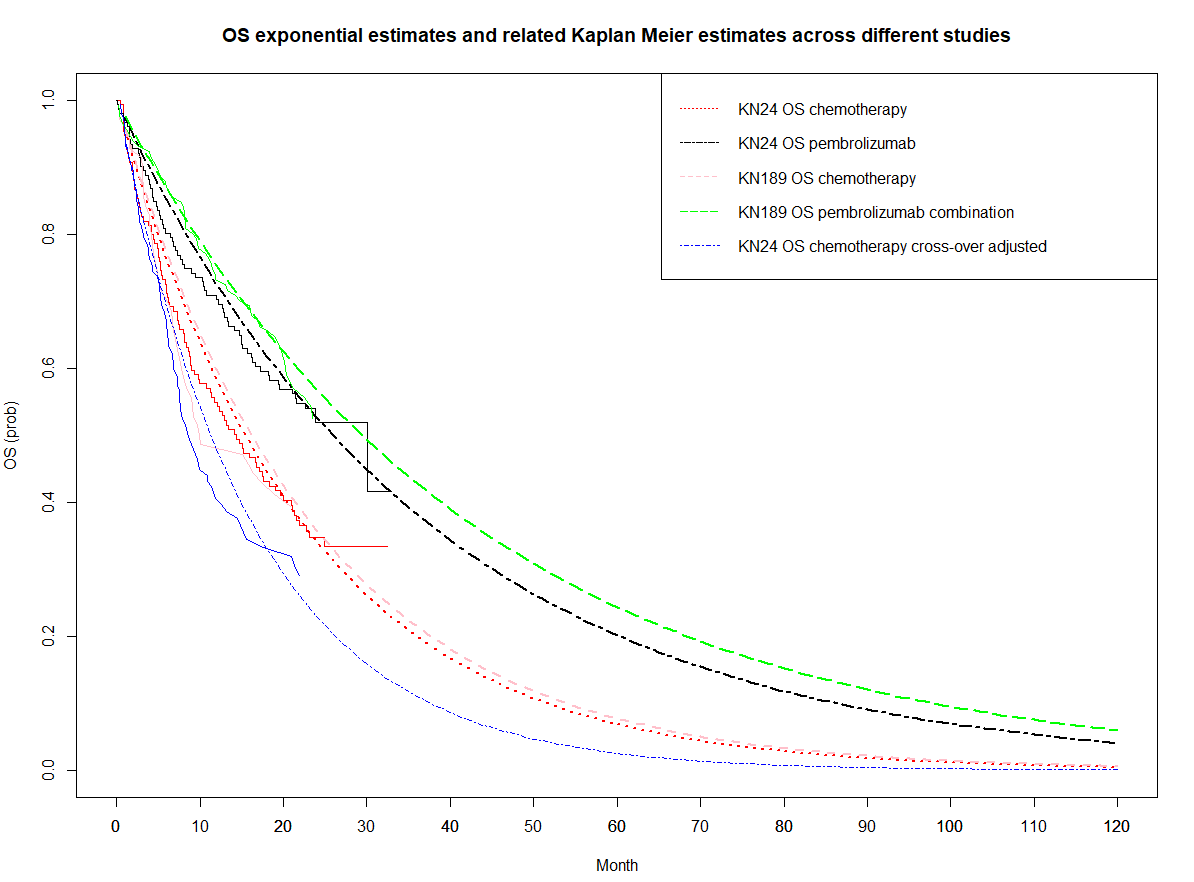


Fig. S2 Modelled overall survival curves. *OS* overall survival

Figure footnote: Dashed lines represent the modelled curves, and undashed lines of the same colour represent the corresponding KM estimates


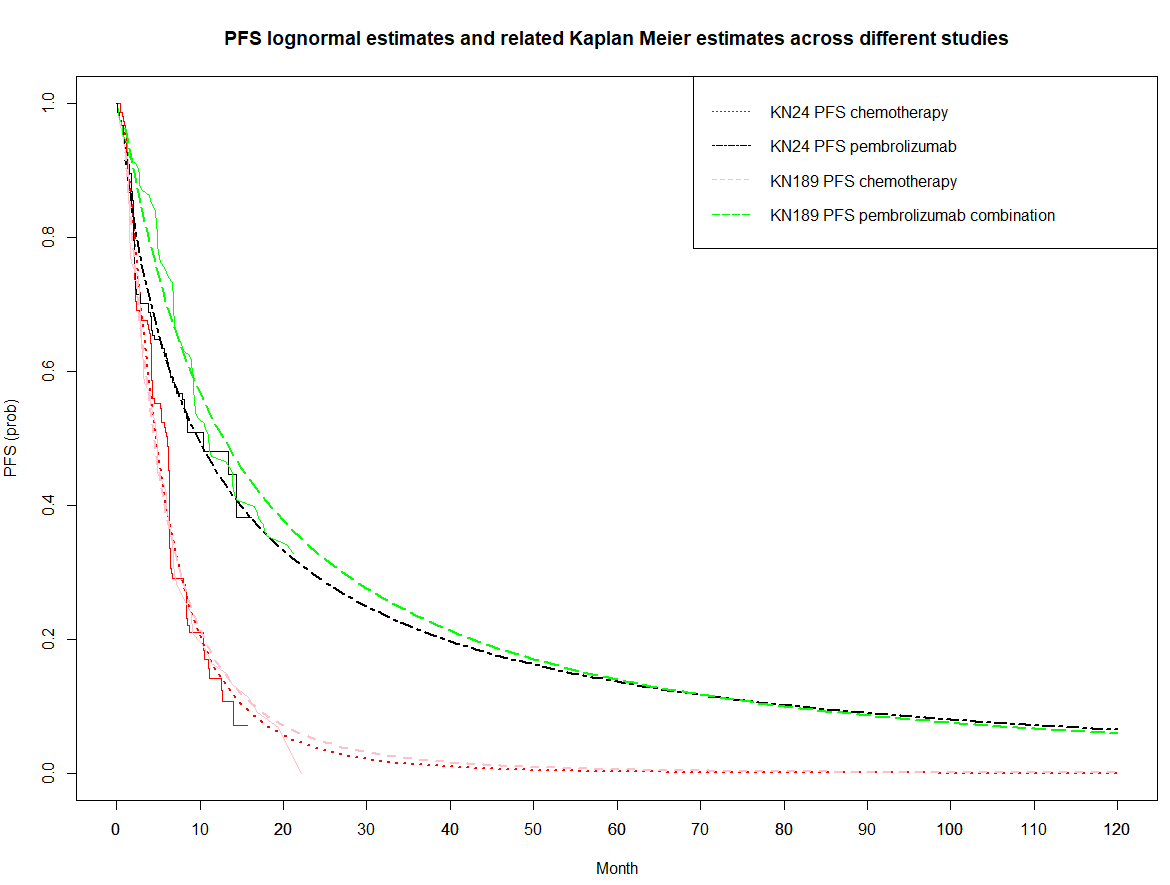


Fig. S3 Modelled progression-free survival curves. *PFS* progression-free survival

Figure footnote: Dashed lines represent the modelled curves, and undashed lines of the same colour represent the corresponding KM estimates.


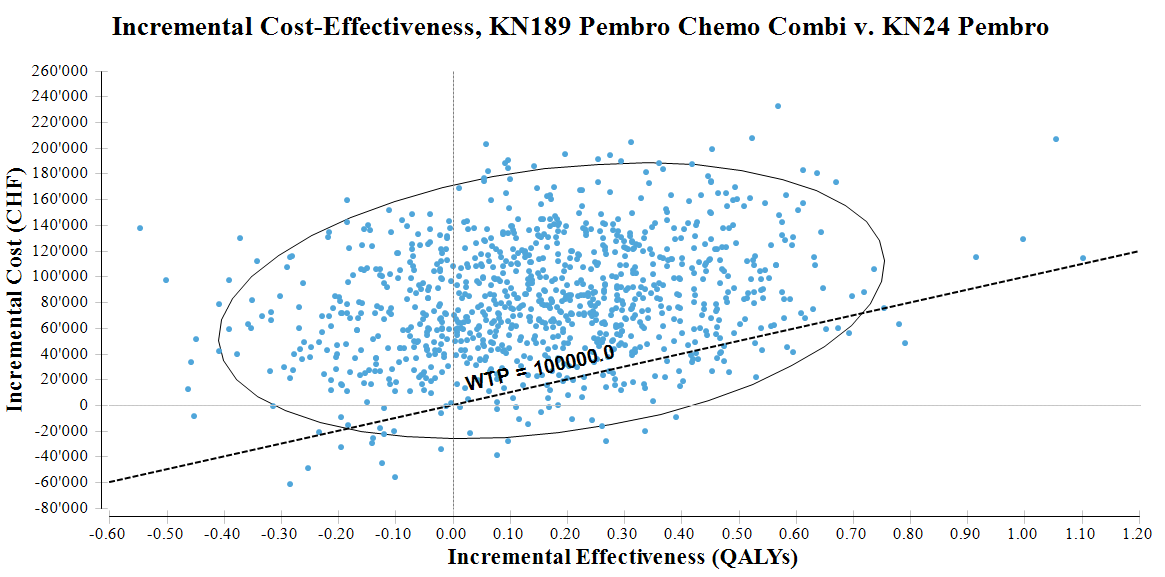


Fig. S4 Cost-effectiveness plane for the probabilistic sensitivity analysis comparing combination therapy to pembrolizumab monotherapy. *CHF* Swiss Franc, *QALYs* quality-adjusted life year, *WTP* willingness-to-pay threshold (CHF per QALY gained)


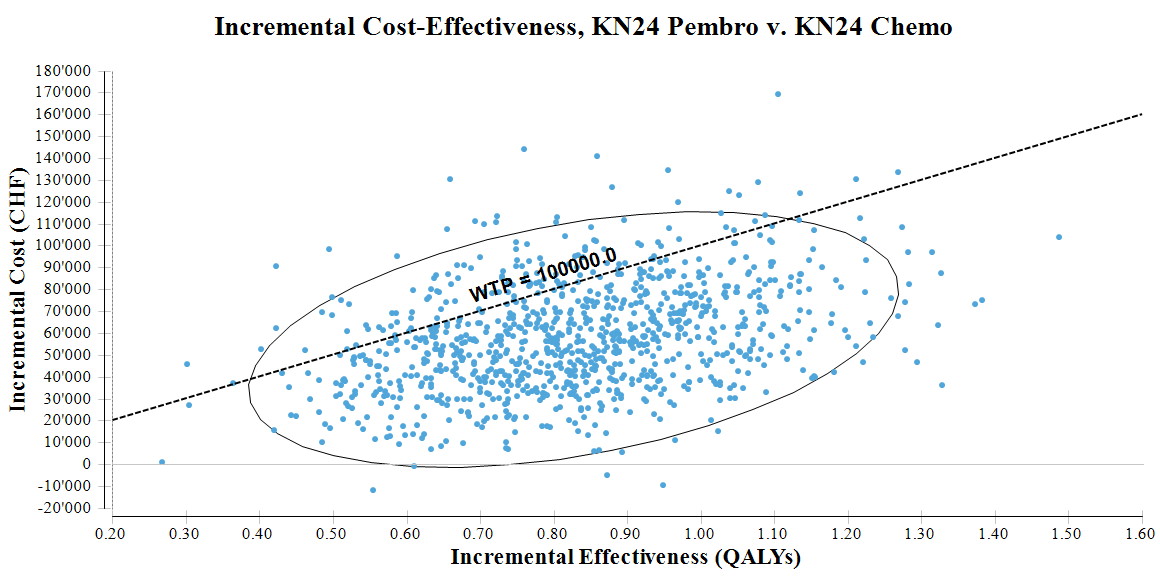


**Fig. S5** Cost-effectiveness plane for the probabilistic sensitivity analysis comparing pembrolizumab monotherapy to chemotherapy. *CHF* Swiss Franc, *QALYs* quality-adjusted life year, *WTP* willingness-to-pay threshold (CHF per QALY gained)


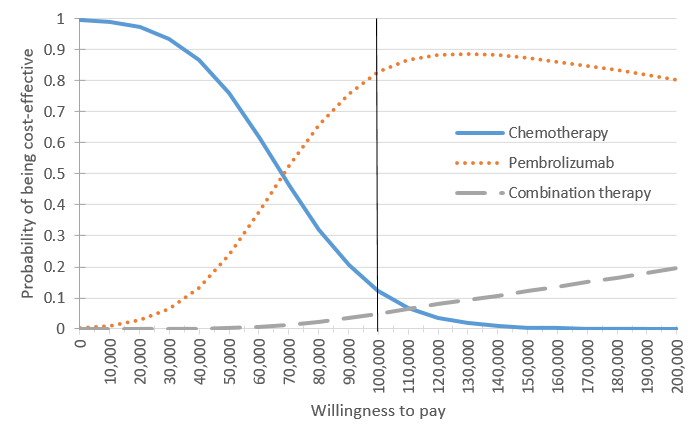


Fig. S6 Cost-effectiveness acceptability curve for the probabilistic sensitivity analysis. *CHF* Swiss Franc, *QALYs* quality-adjusted life year, *WTP* willingness-to-pay threshold (CHF per QALY gained)

**References**

1. Bhadhuri, A., Insinga, R., Guggisberg, P., Panje, C., Schwenkglenks, M.: Cost effectiveness of pembrolizumab vs chemotherapy as first-line treatment for metastatic NSCLC that expresses high levels of PD-L1 in Switzerland. Swiss medical weekly **149**, w20170 (2019). doi:10.4414/smw.2019.20170

2. Borghaei, H., Paz-Ares, L., Horn, L., Spigel, D.R., Steins, M., Ready, N.E., Chow, L.Q., Vokes, E.E., Felip, E., Holgado, E., Barlesi, F., Kohlhaufl, M., Arrieta, O., Burgio, M.A., Fayette, J., Lena, H., Poddubskaya, E., Gerber, D.E., Gettinger, S.N., Rudin, C.M., Rizvi, N., Crino, L., Blumenschein, G.R., Jr., Antonia, S.J., Dorange, C., Harbison, C.T., Graf Finckenstein, F., Brahmer, J.R.: Nivolumab versus Docetaxel in Advanced Nonsquamous Non-Small-Cell Lung Cancer. The New England journal of medicine **373**(17), 1627-1639 (2015). doi:10.1056/NEJMoa1507643

3. Matter-Walstra, K., Schwenkglenks, M., Aebi, S., Dedes, K., Diebold, J., Pietrini, M., Klingbiel, D., von Moos, R., Gautschi, O.: A Cost-Effectiveness Analysis of Nivolumab versus Docetaxel for Advanced Nonsquamous NSCLC Including PD-L1 Testing. Journal of thoracic oncology : official publication of the International Association for the Study of Lung Cancer **11**(11), 1846-1855 (2016). doi:10.1016/j.jtho.2016.05.032

4. Schiller, J.H., Harrington, D., Belani, C.P., Langer, C., Sandler, A., Krook, J., Zhu, J., Johnson, D.H.: Comparison of four chemotherapy regimens for advanced non-small-cell lung cancer. The New England journal of medicine **346**(2), 92-98 (2002). doi:10.1056/NEJMoa011954

5. Gandhi, L., Rodriguez-Abreu, D., Gadgeel, S., Esteban, E., Felip, E., De Angelis, F., Domine, M., Clingan, P., Hochmair, M.J., Powell, S.F., Cheng, S.Y., Bischoff, H.G., Peled, N., Grossi, F., Jennens, R.R., Reck, M., Hui, R., Garon, E.B., Boyer, M., Rubio-Viqueira, B., Novello, S., Kurata, T., Gray, J.E., Vida, J., Wei, Z., Yang, J., Raftopoulos, H., Pietanza, M.C., Garassino, M.C.: Pembrolizumab plus Chemotherapy in Metastatic Non-Small-Cell Lung Cancer. The New England journal of medicine **378**(22), 2078-2092 (2018). doi:10.1056/NEJMoa1801005

6. Herbst, R.S., Baas, P., Kim, D.W., Felip, E., Pérez-Gracia, J.L., Han, J.Y., Molina, J., Kim, J.H., Arvis, C.D., Ahn, M.J., Majem, M., Fidler, M.J., de Castro, G., Jr., Garrido, M., Lubiniecki, G.M., Shentu, Y., Im, E., Dolled-Filhart, M., Garon, E.B.: Pembrolizumab versus docetaxel for previously treated, PD-L1-positive, advanced non-small-cell lung cancer (KEYNOTE-010): a randomised controlled trial. Lancet (London, England) **387**(10027), 1540-1550 (2016). doi:10.1016/s0140-6736(15)01281-7

7. Gadgeel, S.M., Garassino, M.C., Esteban, E., Speranza, G., Felip, E., Hochmair, M.J., Powell, S.F., Cheng, S.Y., Bischoff, H., Peled, N., Hui, R., Reck, M., Kurata, T., Garon, E.B., Boyer, M.J., Yang, J., Pietanza, M.C., Rodriguez-Abreu, D.: KEYNOTE-189: Updated OS and progression after the next line of therapy (PFS2) with pembrolizumab (pembro) plus chemo with pemetrexed and platinum vs placebo plus chemo for metastatic nonsquamous NSCLC. Journal of Clinical Oncology **37**(15_suppl), 9013-9013 (2019). doi:10.1200/JCO.2019.37.15_suppl.9013

8. Guyot, P., Ades, A.E., Ouwens, M.J., Welton, N.J.: Enhanced secondary analysis of survival data: reconstructing the data from published Kaplan-Meier survival curves. BMC medical research methodology **12**, 9 (2012). doi:10.1186/1471-2288-12-9

9. Latimer N: NICE DSU technical support document 14: survival analysis for economic evaluations alongside clinical trials-extrapolation with patient-level data. In. Sheffield, UK: Report by the Decision Support Unit;, (2011)

10. Huang M., C.S., Insinga R., Burke TA., Pellissier J., Pickard AS.,: Health state utilities in metastatic NSCLC: A study of multiple immuno-oncology trials. In: ISPOR Europe, Barcelona, Spain 2018

11. Borget, I., Cadranel, J., Pignon, J.P., Quoix, E., Coudert, B., Westeel, V., Dansin, E., Madelaine, J., Madroszyk, A., Friard, S., Daniel, C., Morin, F., Chouaid, C.: Cost-effectiveness of three strategies for second-line erlotinib initiation in nonsmall-cell lung cancer: the ERMETIC study part 3. The European respiratory journal **39**(1), 172-179 (2012). doi:10.1183/09031936.00201210

12. Lewis, G., Peake, M., Aultman, R., Gyldmark, M., Morlotti, L., Creeden, J., de la Orden, M.: Cost-Effectiveness of Erlotinib versus Docetaxel for Second-Line Treatment of Advanced Non-Small-Cell Lung Cancer in the United Kingdom. Journal of International Medical Research **38**(1), 9-21 (2010). doi:10.1177/147323001003800102

13. Nafees, B., Stafford, M., Gavriel, S., Bhalla, S., Watkins, J.: Health state utilities for non small cell lung cancer. Health and quality of life outcomes **6**, 84 (2008). doi:10.1186/1477-7525-6-84

14. Swiss Federal Office: Swiss Specialty List. <http://www.spezialitätenliste.ch/ShowPreparations.aspx?searchType=SUBSTANCE>. Accessed 22.02.2019

15. TARMED Online Browser: Swiss tariff framework for ambulatory care. <https://www.tarmed-browser.ch/de>.

16. Planchard, D., Popat, S., Kerr, K., Novello, S., Smit, E.F., Faivre-Finn, C., Mok, T.S., Reck, M., Van Schil, P.E., Hellmann, M.D., Peters, S.: Metastatic non-small cell lung cancer: ESMO Clinical Practice Guidelines for diagnosis, treatment and follow-up. Annals of oncology : official journal of the European Society for Medical Oncology **30**(5), 863-870 (2019). doi:10.1093/annonc/mdy474

17. Matter-Walstra, K.W., Achermann, R., Rapold, R., Klingbiel, D., Bordoni, A., Dehler, S., Jundt, G., Konzelmann, I., Clough-Gorr, K.M., Szucs, T.D., Schwenkglenks, M., Pestalozzi, B.C.: Delivery of health care at the end of life in cancer patients of four swiss cantons: a retrospective database study (SAKK 89/09). BMC Cancer **14**, 306 (2014). doi:10.1186/1471-2407-14-306

18. Swiss Federal Statistical Office: Consumer prices. <https://www.bfs.admin.ch/bfs/de/home/statistiken/preise/landesindex-konsumentenpreise.html> (2019). Accessed 19.10.2019

19. Reck, M.e.a.: Keynote-24 3-year survival update: Pembrolizumab versus platinum-based chemotherapy for advanced NSCLC. In: IASLC, World Conference on Lung Cancer, Barcelona, Spain 2019

20. Reck, M., Rodriguez-Abreu, D., Robinson, A.G., Hui, R., Csoszi, T., Fulop, A., Gottfried, M., Peled, N., Tafreshi, A., Cuffe, S., O'Brien, M., Rao, S., Hotta, K., Leiby, M.A., Lubiniecki, G.M., Shentu, Y., Rangwala, R., Brahmer, J.R.: Pembrolizumab versus Chemotherapy for PD-L1-Positive Non-Small-Cell Lung Cancer. The New England journal of medicine **375**(19), 1823-1833 (2016). doi:10.1056/NEJMoa1606774

21. National Institute for Health and Care Excellence: Single Technology Appraisal. Pembrolizumab for treating PD-L1-positive non-small-cell lung cancer after platinum-based chemotherapy [ID840]. In., p. 245. (2016)
